# Genes Involved in the Osteoarthritis Process Identified through Genome Wide Expression Analysis in Articular Cartilage; the RAAK Study

**DOI:** 10.1371/journal.pone.0103056

**Published:** 2014-07-23

**Authors:** Yolande F. M. Ramos, Wouter den Hollander, Judith V. M. G. Bovée, Nils Bomer, Ruud van der Breggen, Nico Lakenberg, J. Christiaan Keurentjes, Jelle J. Goeman, P. Eline Slagboom, Rob G. H. H. Nelissen, Steffan D. Bos, Ingrid Meulenbelt

**Affiliations:** 1 Department of Molecular Epidemiology, Leiden University Medical Center, Leiden, The Netherlands; 2 The Netherlands Genomics Initiative, sponsored by the NCHA, Leiden-Rotterdam, The Netherlands; 3 Department of Pathology, Leiden University Medical Center, Leiden, The Netherlands; 4 Department of Orthopeadics, Leiden University Medical Center, Leiden, The Netherlands; 5 Department of Biostatistics and Bioinformatics, Leiden University Medical Center, Leiden, The Netherlands; University of Pécs Medical School, Hungary

## Abstract

**Objective:**

Identify gene expression profiles associated with OA processes in articular cartilage and determine pathways changing during the disease process.

**Methods:**

Genome wide gene expression was determined in paired samples of OA affected and preserved cartilage of the same joint using microarray analysis for 33 patients of the RAAK study. Results were replicated in independent samples by RT-qPCR and immunohistochemistry. Profiles were analyzed with the online analysis tools DAVID and STRING to identify enrichment for specific pathways and protein-protein interactions.

**Results:**

Among the 1717 genes that were significantly differently expressed between OA affected and preserved cartilage we found significant enrichment for genes involved in skeletal development (e.g. *TNFRSF11B* and *FRZB*). Also several inflammatory genes such as *CD55*, *PTGES* and *TNFAIP6*, previously identified in within-joint analyses as well as in analyses comparing preserved cartilage from OA affected joints versus healthy cartilage were among the top genes. Of note was the high up-regulation of *NGF* in OA cartilage. RT-qPCR confirmed differential expression for 18 out of 19 genes with expression changes of 2-fold or higher, and immunohistochemistry of selected genes showed a concordant change in protein expression. Most of these changes associated with OA severity (Mankin score) but were independent of joint-site or sex.

**Conclusion:**

We provide further insights into the ongoing OA pathophysiological processes in cartilage, in particular into differences in macroscopically intact cartilage compared to OA affected cartilage, which seem relatively consistent and independent of sex or joint. We advocate that development of treatment could benefit by focusing on these similarities in gene expression changes and/or pathways.

## Introduction

Osteoarthritis (OA) is a degenerative disease of the joints causing pain and disability for an increasing proportion of the population thereby imposing a large patient and socio-economic burden [1], [2]. Risk factors for OA include age, sex, joint injury, obesity, and mechanical stresses. In addition, predisposition to OA has a considerable genetic component and it has been proposed that OA can be viewed as a continuum resulting from the interaction between genetics affecting cartilage extracellular matrix composition and joint shape and sensitivity to the other factors mentioned [3], [4]. Major efforts are made to identify loci associated with OA susceptibility to elucidate underlying mechanisms [5]. Treatment options to slow down or reverse the OA process are still very limited and at the time of diagnosis the damage is already irreversible. Together, this emphasizes the importance to increase insight into the disease process and to identify genes and pathways involved in development of OA. A way to achieve this is by investigating the pathophysiological processes in articular cartilage by means of gene expression analyses.

Initially, expression profiles were established for cartilage from knee OA joints in comparison to healthy joints using only a limited number of genes [6]. More recently, exploratory genome wide expression profiling has been performed for the intact cartilage of hip and knee OA joints of patients undergoing joint replacement surgery compared to non-OA joints either derived from autopsies or from neck of femur fractures [7], [8]. These studies showed that many genes involved in extracellular matrix (ECM) production as well as genes involved in ECM degradation or in inflammation were changed. Together, this resulted in significant enrichment for genes involved in skeletal development and response to external stimuli. Although studies that compare healthy cartilage with the preserved cartilage of joints from OA patients are very useful to acquire insight into the pathogenetic differences, the findings are likely biased by confounding factors such as innate differences, age, and stratification by joint. Moreover, due to the study design distinction between age-related changes and early or late changes of OA pathophysiology is hampered.

One of the characteristics of OA is focal loss of articular cartilage, resulting in areas of degradation as well as areas with a relative preservation of cartilage thickness and appearance in the joint. Insight into gene expression specific for the focal areas of cartilage degradation compared to those in preserved areas can provide clues towards dynamic changes of genes and pathways involved in OA pathophysiology independent of confounding factors such as age. Gene expression profiles of cartilage from OA affected and macroscopically preserved areas of the same joint have been determined before, however, in most of these studies limited numbers of donors (4–5 knee joints) were included [9]–[11].

As part of the ongoing Research Arthritis and Articular Cartilage (RAAK) study we set out to perform genome wide analysis of differential gene expression by comparing 33 pairs of matched OA affected and preserved cartilage samples, originating from the same joint of patients that underwent total joint replacement of either hip or knee. Results provide further insights in the ongoing OA disease processes in cartilage, in particular into differences in macroscopically intact cartilage compared to OA affected cartilage.

## Materials and Methods

### Ethics statement

Participants of the RAAK study provided written informed consent. The ongoing RAAK study and its consent procedure is approved by the institutional ethics review committee (Commissie Medische Ethiek of the Leiden University Medical Center; protocol no. P08.239).

### Discovery cohort

The RAAK study is aimed at the biobanking of joint materials as well as mesenchymal stem cells and primary chondrocytes from patients and controls in the Leiden University Medical Center and collaborating outpatient clinics in the Leiden area. In the current study we used paired preserved and OA affected cartilage samples for 33 donors undergoing joint replacement surgery for primary OA (22 hips, 11 knees). Characteristics of the donors are shown in Table S1.

At the moment of collection (within 2 hours following surgery) tissue was washed extensively with phosphate buffered saline (PBS) to decrease the risk of contamination by blood. Cartilage was classified macroscopically and collected separately from OA affected and preserved regions around the weight-bearing area of the joint (Figure S1). Classification was done based on predefined features of OA related damage as described previously [9], [10]: color/whiteness of the cartilage, surface integrity as determined by visible fibrillation/crack formation, and depth and hardness of the cartilage upon sampling with a scalpel. Care was taken to avoid contamination with bone or synovium. Collected cartilage was snap frozen in liquid nitrogen and stored at −80°C prior to RNA extraction.

### Validation and replication cohort

Validation was performed by RT-qPCR in 8 sample pairs of the discovery cohort (3 knee and 5 hip) and for replication of the results we included 28 additional matched sample pairs (20 knee, 8 hip) of similar mean age (shown in Table S1). Sampling procedures were according to the discovery cohort.

### RNA isolation

Cartilage samples were pulverized using a Retsch MM200 under cryogenic conditions. On average 150 mg of pulverized cartilage was dissolved in 1 ml of Trizol reagent, and mixed vigorously. After addition of 200 µl of chloroform the sample was mixed and centrifuged for 15 minutes (16,000 g). The clear aqueous layer was transferred to a new vial and 1 volume of 70% ethanol/DEPC-treated water was added to precipitate RNA. RNA was collected using Qiagen mini columns according to the manufacturers protocol and quality was assessed using a Bioanalyzer lab-on-a-chip. RNA integrity numbers above 8 were considered suitable for microarray analysis.

### Microarrays

After *in* *vitro* transcription, amplification, and labeling with biotin-labeled nucleotides (Illumina TotalPrep RNA Amplification Kit) Illumina HumanHT-12 v3 microarrays were hybridized. Sample pairs were randomly dispersed over the microarrays, however each pair was measured on a single chip. Microarrays were read using an Illumina Beadarray 500GX scanner and after basic quality checks using Beadstudio software data were analyzed in R statistical programming language. Intensity values were normalized using the “rsn” option in the Lumi-package and absence of large scale between-chip effects was confirmed using the Globaltest-package in which the individual chip numbers were tested for association to the raw data [12]. After removal of probes that were not reliably detected (detection *P*>0.05 in more than 50% of the samples) a paired t-test was performed for the remaining 13277 probes comprising 11421 unique genes on all sample pairs while adjusting for chip (to adjust for possible batch effects) and using multiple testing correction as implemented in the “BH” (Benjamini and Hochberg) option in the Limma-package. Analyses for differential expression between OA and healthy and between preserved and healthy cartilage was performed likewise, adjusting in addition for sex and for age.

Gene expression profiles of the samples have been deposited in NCBI’s Gene Expression Omnibus [13] and are accessible through GEO Series accession number GSE57218.

### Quantitative reverse transcription PCR (RT-qPCR)

0.5 µg of total RNA was processed with the First Strand cDNA Synthesis Kit according to the manufacturer’s protocol (Roche Applied Science) and RT-qPCR was performed for the 19 genes showing at least 2-fold expression differences in the microarray analysis (Taqman gene expression assays used are listed in Table S2) using the Biomark 96.96 Dynamic Arrays Fluidigm RT-qPCR platform [14]. Relative gene expressions were calculated with the 2^−ΔΔCt^ method [15], using household gene Beta Actin (*ACTB*) expression as internal standard.

### Immunohistochemistry and staining analysis

For histological examination, joints were fixed using a 4% formaldehyde solution. Subsequently, samples were decalcified using a 10% EDTA solution and embedded in paraffin. Sections (5 µm) adjacent to the collected area were stained using hematoxilin and eosin (H&E) and toluidine blue. Immunohistochemistry was performed for SERPINE1 (mouse monoclonal antibody from American Diagnostica Inc.) without antigen retrieval and for CD55 (rabbit polyclonal antibody from Santa Cruz Biotechnology Inc.) with heat antigen retrieval (0,01 M Citratebuffer pH = 6.0) as described previously [16].

Quantification of OA related cartilage damage was scored by 2 observers (JVMGB and YFMR) according to Mankin *et al* [17]. Quantification of SERPINE1 expression was performed by scoring staining of chondrocytes in the superficial, middle, and deep cartilage layer with a score of 0 (no staining), 1 (moderate staining), or 2 (strong staining). Using Generalized Estimating Equations, scores were summed and used as a predictor variable with Mankin score as outcome whilst correcting for sex and age of each donor.

### Pathway analysis and protein-protein interaction

Gene enrichment among the 1717 genes showing significant differential expression was performed with the Database for Annotation, Visualization and Integrated Discovery (DAVID) tool [18] selecting for biological processes identified in the Gene Ontology database (GOTERM_BP_FAT in the options menu implemented in DAVID), selecting for cell compartment (GOTERM_CC_FAT), or selecting for molecular function (GOTERM_MF_FAT) and using the microarray background (HumanHT-12_V3_0_R2_11283641_A). Pathways with *P*≤0.05 after correction for multiple testing according to Bonferroni were considered significant (Bonferroni corrections were performed by multiplying the raw P-values with the number of genes included in the analysis).

Enrichment in protein-protein interactions among the significant genes was analyzed with the Search Tool for the Retrieval of Interacting Genes/Proteins (STRING) 9.0 [19] available online.

### Literature based candidate genes

Based on selected publications of genome wide association study meta-analyses [20]–[29] we investigated our expression dataset for evidence of differential gene expression of the reported significant candidate genes.

## Results

### Differential expression between preserved and OA affected cartilage

To identify genes with changed expression in response to ongoing OA processes genome wide expression profiles were generated for preserved and OA affected cartilage of the same joint of 33 donors. Characteristics of the donors are shown in Table S1. Males (N = 13) and females (N = 20) included in the study were aged between 54 and 80 years (mean age: 66.2). In total, 22 patients received a hip replacement and 11 patients underwent total knee replacement. Among all OA joints included in this study (61 in total), 28 pairs were randomly selected to assess the Mankin scores of preserved and affected areas. Mankin scores were significantly higher in the samples macroscopically designated as ‘OA affected’ as compared to sections distinguished as ‘preserved’ (mean Mankin score 7.8 vs. 4.7, respectively, *P* = 4×10^−4^, paired t-test) and as a result gene expression differences can be directly linked to these differences in Mankin scores.

After normalization and correction for multiple testing, significant differential expression between the OA affected and preserved cartilage was identified for 1893 probes, representing 1717 unique genes (Table S3). Among the 1717 unique genes 19 were differently expressed with fold-changes of 2 and higher (Table 1). Notably, 14 of these were up-regulated in OA as compared to preserved cartilage and only 5 were down-regulated. Overall, 748 (44%) of the differentially expressed genes were up-regulated. Larger fold changes were observed in expression of genes well known for their association with OA cartilage such as tumor necrosis factor alpha-induced protein 6 (*TNFAIP6* also known as *TSG-6*, 2.9-fold up in OA cartilage; *P* = 4.4×10^−8^), cytokine receptor-like factor 1 (*CRLF1*, 3-fold up in OA cartilage; *P* = 4.4×10^−8^), and *Wnt*-inhibitor frizzled related protein beta (*FRZB*, 2.5-fold down in OA cartilage; *P* = 1.3×10^−6^). A notable gene highly up-regulated in OA cartilage was neuronal growth factor (*NGF*, 2.3-fold up; *P* = 3.4×10^−7^).

**Table 1:** Genes significantly differently expressed in OA affected cartilage.

		Discovery					Replication		
Gene symbol	Function	FC	Dir.	Pval			FC	Dir.	Pval
*TNFAIP6*	cartilage development	2.90	Up	4.40×10^−8^			4.01	Up	3.61×10^−4^
*CRLF1*	immune response	2.97	Up	4.40×10^−8^			4.01	Up	8.09×10^−6^
*LEPREL1*	cartilage development	2.37	Up	4.40×10^−8^			2.88	Up	9.49×10^−4^
*CD55*	complement cascade	2.39	Up	4.40×10^−8^			3.00	Up	5.18×10^−5^
*RARRES2*	immune response	2.26	Down	6.06×10^−8^			3.32	Down	1.23×10^−4^
*NGF*	neurogenesis	2.26	Up	3.36×10^−7^			4.54	Up	2.50×10^−5^
*FRZB*	bone development	2.54	Down	1.28×10^−6^			3.04	Down	4.39×10^−4^
*SERPINE1*	complement cascade	2.40	Up	1.30×10^−6^			3.13	Up	1.54×10^−4^
*PTGES*	immune response	2.34	Up	1.46×10^−6^			3.29	Up	1.76×10^−5^
*SPP1*	ECM-receptor interaction	2.53	Up	3.40×10^−6^			2.56	Up	7.05×10^−3^
*CXCL14*	immune response	2.16	Up	3.54×10^−6^			4.97	Up	1.01×10^−4^
*TNFRSF11B*	bone development	2.10	Up	4.23×10^−6^			2.69	Up	6.58×10^−4^
*FN1*	ECM-receptor interaction	2.31	Up	1.13×10^−5^			1.52	Up	3.28×10^−2^
*HBA2*	oxygen transport	2.26	Up	2.02×10^−5^			2.97	Up	6.15×10^−3^
*HBB*	oxygen transport	2.00	Up	6.97×10^−5^			2.50	Up	1.69×10^−2^
*PAPPA*	wound healing	2.08	Up	7.69×10^−5^			2.25	Up	4.05×10^−3^
*CRISPLD1*	–	2.50	Down	1.42×10^−4^			1.05	–	7.54×10^−1^
*COL9A1*	cartilage development	2.18	Down	2.67×10^−4^			3.14	Down	2.27×10^−4^
*CHRDL2*	cartilage development	2.39	Down	3.55×10^−4^			5.33	Down	5.71×10^−4^

Shown are results of the microarray analysis (Discovery) and the replication for genes with at least 2-fold change (Dir.: direction of effects relative to OA; FC: fold change; Pval: P-value).

Validation of the 19 genes with fold-changes of 2 or higher in 8 sample pairs used in the microarray analyses by means of RT-qPCR showed similar effect sizes and directions as those found in the microarray analysis (Table S5). Replication performed in an additional set of 28 independent preserved and affected cartilage sample pairs also showed comparable effect sizes and directions and, except for cysteine-rich secretory protein LCCL domain-containing 1 precursor (*CRISPLD1*), all genes were significantly different expressed (Table 1). Individual expression boxplots of the replicated genes are shown in Figure S2.

Expression profiles of genes with fold-changes of 2 and higher were analyzed for association with Mankin score as a grade of disease severity (Table 2). Almost all genes associated with Mankin score, except for *COL9A1*, *HBA2* and *HBB*. To further characterize expression of the 19 genes with highest fold changes in OA affected cartilage, we investigated whether the observed changes were either joint or sex specific. As shown in Table 2, for most of the genes fold changes of the (joint or sex) stratified analyses were highly comparable and not statistically different from those of the discovery analysis. However, increased expression of pregnancy-associated plasma protein A (*PAPPA*) was significantly less pronounced in knee OA (1.3-fold increase) than in hip OA (2.6-fold increase).

**Table 2:** Characteristics of the replicated genes.

		Hip		Knee	Male		Female		
Gene symbol	pVal_Mankin_	FC	pVal	FC	pVal	FC	pVal	FC	pVal
*TNFAIP6*	1.E-05	2.80	3.89E-05	3.10	6.19E-03	3.09	7.88E-04	2.78	2.11E-04
*CRLF1*	3.E-07	2.93	1.49E-04	3.06	1.47E-03	3.51	1.22E-03	2.67	2.34E-04
*LEPREL1*	5.E-15	2.50	3.85E-05	2.12	1.67E-02	2.90	7.88E-04	2.07	2.52E-04
*CD55*	8.E-11	2.67	3.85E-05	1.92	6.86E-03	2.63	1.53E-03	2.25	2.34E-04
*RARRES2*	6.E-08	0.42	3.85E-05	0.48	3.18E-02	0.44	5.51E-03	0.44	2.11E-04
*NGF*	2.E-09	2.39	1.49E-04	2.03	7.26E-03	2.68	5.48E-03	2.02	2.69E-04
*FRZB*	1.E-05	0.35	1.30E-04	0.50	3.67E-02	0.36	3.20E-03	0.41	1.95E-03
*SERPINE1*	1.E-03	2.40	6.10E-04	2.41	6.19E-03	2.64	8.36E-03	2.26	1.15E-03
*PTGES*	5.E-05	2.40	3.54E-04	2.21	1.70E-02	2.81	5.05E-03	2.07	1.84E-03
*SPP1*	3.E-05	2.56	1.94E-04	2.46	5.87E-02	2.64	9.09E-03	2.45	2.36E-03
*CXCL14*	2.E-04	2.49	1.87E-04	1.63	3.16E-02	2.73	9.09E-03	1.85	1.29E-03
*TNFRSF11B*	1.E-05	2.27	1.87E-04	1.80	8.65E-02	2.63	8.10E-03	1.82	2.71E-03
*FN1*	9.E-05	2.64	1.49E-04	1.83	1.40E-01	2.89	9.35E-03	2.00	4.48E-03
*HBA2*	1.E-01	2.46	1.01E-03	1.91	5.04E-02	1.96	2.46E-02	2.49	2.87E-03
*HBB*	2.E-01	2.17	1.50E-03	1.70	1.30E-01	1.72	2.86E-02	2.21	5.56E-03
*PAPPA*	2.E-05	2.59	4.68E-04	1.34	1.98E-01	2.22	4.79E-02	1.99	6.10E-03
*CRISPLD1*	6.E-02	0.38	5.98E-03	0.44	4.28E-02	0.36	2.56E-03	0.43	3.47E-02
*COL9A1*	6.E-01	0.42	6.24E-03	0.54	6.46E-02	0.42	4.95E-03	0.49	4.52E-02
*CHRDL2*	3.E-04	0.37	3.56E-03	0.52	2.09E-01	0.34	2.70E-02	0.48	3.50E-02

Results of the analysis for association of the genes with Mankin score and for joint- and sex-stratified analyses.

In addition to the gene expression profiles of preserved and OA affected cartilage, gene expression profiles were also generated for 7 healthy cartilage (characteristics of the donors are shown in Table S1) and explored for the 19 genes. For most of these 19 genes we did not find significant differences between healthy and preserved cartilage. However, when analyzing the trend of the differences between healthy, preserved and OA affected cartilage we did find a significant linear effect on the expression of most genes. In contrast, expression changes of *CRISPLD1* and *COL9A1* in healthy versus preserved cartilage were not significant and appeared to be increased while the expression in preserved versus OA affected cartilage was found to be decreased (Figure S3).

### Functional annotation of genes differently expressed in OA affected cartilage

To investigate whether the genes differently expressed between preserved and OA affected cartilage belonged to specific pathways, we used the online functional annotation tool DAVID. Seven GO-terms referring to 6 independent pathways were identified (Table 3). The most significant GO-term was observed for “skeletal system development”. This term captured several of the genes with fold-changes of 2 or higher (e.g. *FRZB* and *TNFRSF11B*). Furthermore, of note was the GO-term referring to “extracellular matrix organization” including decorin (*DCN*) and several collagens (e.g. *COL1A2*, *COL2A1*, *COL3A1*). When analyzing for enrichment using the cellular compartment option, most significant GO-term was “extracellular matrix”, and analyzing for molecular function showed genes involved in “copper ion binding” and “glycosaminoglycan binding” to be significantly enriched (Table 3).

**Table 3:** Gene enrichment analysis in OA affected versus preserved cartilage.

Analysis option:	GO-Term	Count	Pct.	Enr.	Pval	Pval_adj_	FDR
Biological Processes	GO:0001501∼skeletal system development	59	3.86	2.12	5.21×10^−8^	7.97×10^−5^	9.53×10^−5^
Biological Processes	GO:0007167∼enzyme linked receptor protein signaling pathway	56	3.66	1.88	5.74×10^−6^	8.78×10^−3^	1.05×10^−2^
Biological Processes	GO:0008285∼negative regulation of cell proliferation	57	3.73	1.81	1.52×10^−5^	2.32×10^−2^	2.77×10^−2^
Biological Processes	GO:0005996∼monosaccharide metabolic process	40	2.61	2.07	1.64×10^−5^	2.51×10^−2^	3.00×10^−2^
Biological Processes	GO:0030198∼extracellular matrix organization	24	1.57	2.67	2.10×10^−5^	3.22×10^−2^	3.85×10^−2^
Biological Processes	GO:0007155∼cell adhesion	93	6.08	1.53	3.11×10^−5^	4.76×10^−2^	5.69×10^−2^
Biological Processes	GO:0022610∼biological adhesion	93	6.08	1.53	3.30×10^−5^	5.06×10^−2^	6.04×10^−2^
Cellular Compartment	GO:0031012∼extracellular matrix	69	4.51	2.26	1.49E-10	2.29E-07	2.19E-07
Cellular Compartment	GO:0005578∼proteinaceous extracellular matrix	65	4.25	2.03	2.63E-10	4.02E-07	3.86E-07
Cellular Compartment	GO:0044421∼extracellular region part	133	8.69	1.56	1.10E-07	1.68E-04	1.61E-04
Cellular Compartment	GO:0044420∼extracellular matrix part	30	1.96	2.90	2.16E-07	3.31E-04	3.17E-04
Cellular Compartment	GO:0005581∼collagen	14	0.92	4.45	5.20E-06	7.95E-03	7.62E-03
Molecular Function	GO:0005507∼copper ion binding	19	1.24	3.36	6.69E-06	1.02E-02	1.08E-02
Molecular Function	GO:0005539∼glycosaminoglycan binding	29	1.90	2.40	1.99E-05	3.04E-02	3.20E-02
Molecular Function	GO:0005198∼structural molecule activity	84	5.49	1.58	2.52E-05	3.85E-02	4.06E-02

Analysis considering the biological processes option in DAVID (GOTERM_BP_FAT), the cellular compartment option (GOTERM_CC_FAT), or the molecular function option (GOTERM_MF_FAT) as indicated in the first column, using medium classification stringency for all genes significantly differently expressed between OA affected and preserved cartilage (GO-Term: GO-terms within the different clusters; Count: number of genes identified for the respective GO-term; Pct: percentage of genes from total number of genes tested; Enr.: fold enrichment of indicated pathway; Pval: P-value; Pval_adj_: adjusted P-value; FDR: false discovery rate).

**Figure 1:**
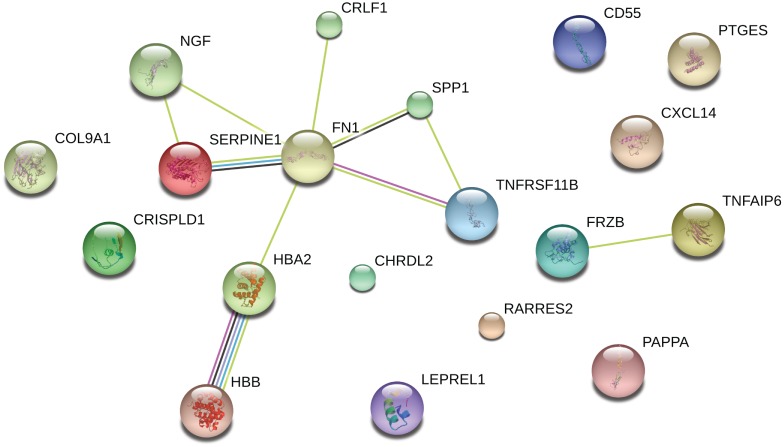
Protein-protein interaction between the genes with expression changes of at least 2-fold (Table 1) as determined with STRING.

Among the 19 genes that were highly changed in OA affected cartilage (at least 2-fold), 3 pathways were significantly enriched with lowest P-value for GO-term “response to wounding” (Table 4), which included the genes *TNFAIP6*, *SERPINE2*, and *CD55*. When analyzing for interaction among proteins encoded by these genes using STRING, we found significant enrichment for protein-protein interactions (*P* = 4.7×10^−10^) in which fibronectin-1 (*FN1*) seemed to play a central role considering that 6 of the 19 proteins were found to relate to *FN1* (Figure 1).

**Table 4:** Gene enrichment analysis.

Term	Count	Pct	Enr.	Pval	Pval_adj_	FDR
GO:0009611∼response to wounding	6	31.58	8.87	2.96E-04	5.63E-03	3.94E-01
GO:0001501∼skeletal system development	5	26.32	12.10	4.95E-04	9.40E-03	6.58E-01
GO:0006954∼inflammatory response	5	26.32	12.06	5.01E-04	9.51E-03	6.65E-01

Pathway analysis considering the biological processes option in DAVID (GOTERM_BP_FAT) using the genes from Table 1 with at least 2-fold expression difference between OA affected and preserved cartilage (GO-Term: GO-terms within the different clusters; Count: number of genes identified for the respective GO-term; Pct: percentage of genes from total number of genes tested; Enr.: fold enrichment of indicated pathway; Pval: P-value; Pval_adj_: adjusted P-value; FDR: false discovery rate).

### Immunohistochemical assessment of proteins encoded by genes identified in the microarray analysis

**Figure 2:**
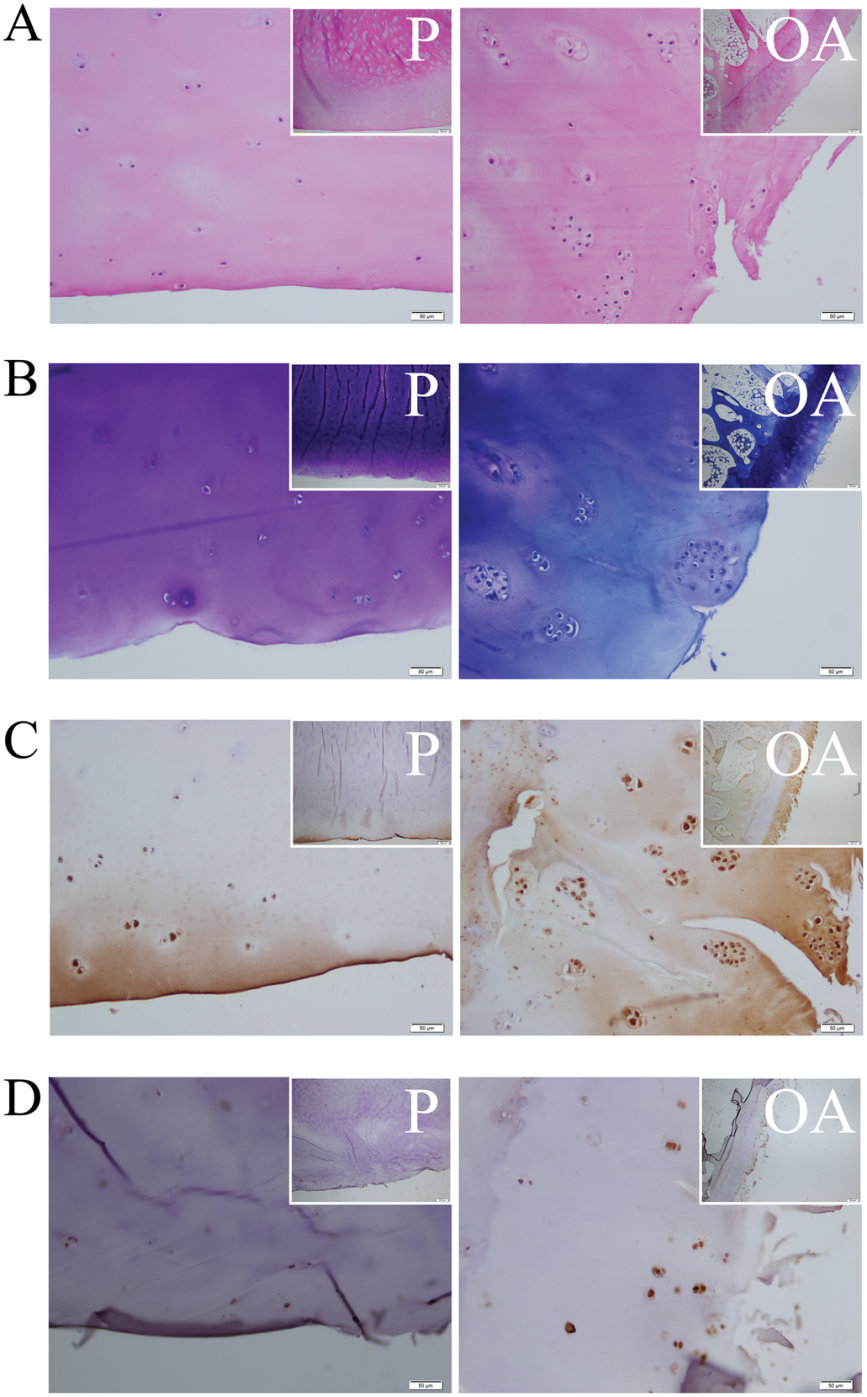
Representative slides of immunohistochemical staining. A) H&E staining. B) Toluidine blue staining. C) SERPINE1. D) CD55 (magnification 20x; insets show larger overview at magnification 4x; white scale bars indicate 50 µm and 200 µm, respectively). The left panels show preserved cartilage area (P) and the right panels show the OA affected cartilage area (OA).

In addition to differential expression of proteins encoded by genes found in the microarray analysis, immunohistochemical (IHC) staining provides insight in expression pattern and localization of differentially expressed genes in the different cartilage layers. Therefore, as a proof of principal, IHC was performed for SERPINE1 and CD55. Figure 2 shows representative sections for the staining of preserved (P) versus OA affected cartilage, with Figure 2A and B showing an example of the H&E and Toluidine blue staining respectively (upper panel: 4x magnification, lower panel 20x magnification).

Immunohistochemistry for SERPINE1 showed that the protein is expressed in chondrocytes and differential expression between OA and preserved cartilage at the protein level was very pronounced (Figure 2C). In OA affected cartilage, SERPINE1 was not only expressed in the superficial layer, but also in the middle layer. In the most affected parts, we even observed SERPINE1 protein expression in chondrocytes residing in the deep zone. In addition, increasing matrix staining of SERPINE1 was observed with increasing OA affection state. We performed a quantification of the staining as described in Materials and Methods, and statistical analysis showed a significant difference between protein abundance in OA and preserved cartilage (*P* = 2.4×10^−4^). The expression difference seemed to correlate mostly with toluidine blue staining, and thus with the level of proteoglycan constituents of chondromucin aggregates in the samples (*P* = 2.9×10^−9^).

CD55 protein expression was most pronounced in the superficial layer, with higher levels in the more OA affected zones of the cartilage, while hardly any CD55 positive cells were detected in the deep layer (Figure 2D). The differences, however, were more subtle than for SERPINE1 and the range in the quantification did not allow for statistical analysis.

### Prioritization of genes residing in compelling genome wide association signals

In order to explore whether genes identified by genome wide association studies are active in cartilage and/or change in response to the OA process, we screened for differential expression of genes originating from recently published large scale meta analyses on OA (Table 5). Sixteen of the 29 genes selected were well detected in the microarray (*P*_detection_≤0.05) and from these, 8 were significantly different between OA and preserved cartilage. Most genes showed only modest expression changes. Of note was differential expression of the HMG-box transcription factor 1 (*HBP1*) gene, identified in the Rotterdam study [24], which showed 1.1-fold up-regulation in OA cartilage (*P* = 2.0×10^−3^).

**Table 5:** Genes identified in robust genome wide approaches with fold-changes and P-values for OA versus preserved cartilage (OA vs P).

			OA vs P	
Gene	Ref	Joint published	FC	Pval
*ASTN2*	[28]	Hip&Knee	–	–
*BCAP29*	[24]	Knee	1.1	1.7×10^−2^
*BTNL2*	[26]	Knee	–	–
*C6ORF130*	[27]	Hip&Knee	0.88	1.6×10^−3^
*CDC5L*	[28]	Hip&Knee	–	–
*CHST11*	[28]	Hip&Knee	–	–
*COG5*	[24]	Knee	0.98	2.1×10^−1^
*COL11A1*	[27]	Hip&Knee	0.94	4.5×10^−1^
*DOT1L*	[20]	Hip	–	–
*DUS4L*	[24]	Knee	–	–
*DVWA*	[25]	Knee	–	–
*FILIP1*	[28]	Hip&Knee	–	–
*FTO*	[28]	Hip&Knee	1.0	8.5×10^−1^
*GDF5*	[21]	Hip&Knee	1.1	4.3×10^−2^
*GLT8D1*	[28]	Hip&Knee	1.0	3.0×10^−1^
*GNL3*	[28]	Hip&Knee	1.1	4.6×10^−2^
*GPR22*	[24]	Knee	–	–
*HBP1*	[24]	Knee	1.1	2.0×10^−3^
*HLA-DQB1*	[26]	Knee	–	–
*KLHDC5*	[28]	Hip&Knee	1.0	4.8×10^−1^
*MCF2L*	[22]	Hip&Knee	–	–
*MICAL3*	[27]	Hip&Knee	–	–
*NCOA3*	[23]	Hip	0.93	7.9×10^−3^
*PAPPA*	[28]	Hip&Knee	2.1	1.1×10^−6^
*PRKAR2B*	[24]	Knee	1.0	9.9×10^−1^
*PTHLH*	[28]	Hip&Knee	1.4	1.8×10^−3^
*SENP6*	[28]	Hip&Knee	1.1	3.3×10^−1^
*SUPT3H*	[28]	Hip&Knee	1.0	6.1×10^−1^
*TP63*	[28]	Hip&Knee	–	–
*VEGF*	[29]	Hip	1.0	4.5×10^−1^

(Ref: reference, where indicated gene was published as OA susceptibility gene; Pval: nominal P-value; FC: fold change; –: not detected on microarray).

## Discussion

As part of the RAAK study we compared genome wide expression levels between preserved and OA affected cartilage of the same joint from 33 donors. Such a paired study design allows the detection of genes specifically involved in the OA pathophysiological process, independent of inter-individual or age-related confounding factors as also reflected by the highly comparable differential gene expression patterns when stratifying according to joint and sex. After correction for multiple testing 1717 unique genes showed significant differential expression, of which 19 genes had a fold-change of 2 or higher. In an independent paired cartilage sample set, differential expression was confirmed for 18 genes by RT-qPCR. For most of these genes, except *HBA2*, *HBB*, and *COL9A1*, expression associated with disease severity as determined by scoring according to Mankin [17], and OA-associated increase in protein expression for 2 genes (*CD55* and *SERPINE1*) was demonstrated by immunohistochemistry.

We confirmed several genes previously identified in within-joint analyses for OA affected versus preserved cartilage as well as analyses comparing preserved cartilage from OA affected joints versus healthy cartilage such as the inflammatory genes *CD55* [8], *PTGES* and *TNFAIP6* [11]. This overlap is noteworthy since in our analysis considerably more samples were included. A large sample size increases power to detect replicable findings and allows detection of differences that were previously missed or more subtle. Our data thus indicate that at least a number of genes are consistently involved in the OA disease process despite the appreciated heterogeneous pathophysiology. Another gene present among the top genes and highly up-regulated in OA affected cartilage was the tumor necrosis factor receptor superfamily 11b (*TNFRSF11B*) gene encoding osteoprotegerin. Very recently we reported in this protein on a gain of function mutation likely causal in a family with early onset OA with chondrocalcinosis [30]. In this respect, the up-regulated expression, could contribute to respective mineralization of the cartilage and eventually formation of bone, a major hallmark of the ongoing osteoarthritis disease process.

Studies comparing intact cartilage with OA affected cartilage of the same joint allow detection of gene expression changes specific to the ongoing OA pathophysiological processes independent of confounding factors such as sex and age and joint as was also demonstrated by the highly comparable results of our stratified analysis. Identification of such genes commonly changing during OA independent of joint site or sex could be very useful with respect to drug development. On the other hand, differences identified between the intact cartilage derived from patients undergoing joint replacement surgery and healthy cartilage of independent joints are of a cross-sectional nature and provide information on innate differences among OA patients as well as genes changing during OA. Therefore, genes overlapping among the different studies may be of interest to better understand dynamic changes during onset and ongoing OA. A notable example was the expression of the *COL9A1* gene that was higher in preserved as compared to healthy cartilage (3.6-fold), but was subsequently decreased in the OA affected cartilage (Figure S3). Although we acknowledge the fact that the included 7 healthy cartilage samples had a large age-range, our results are in line with the findings of Karlsson *et al* [8] and Xu *et al* [7] showing increased expression of *COL9A1* in cartilage from patients undergoing joint replacement surgery in comparison to healthy cartilage. This altered direction of effect in ongoing OA may explain the fact that *COL9A1* was found not to be associated with Mankin score and suggests that it is mainly involved in the initial response of the chondrocyte to cartilage damage. Gene enrichment analyses performed with all significant genes showed especially that genes involved in the skeletal development were changed in OA affected as compared to preserved cartilage. Notably, this is in accordance with observations from Xu *et al* [7] who found enrichment of genes involved in skeletal development by comparing healthy cartilage versus cartilage of OA affected joints, suggesting that this is a pathway commonly affected in OA cartilage, both in the initiation phases as well as in ongoing OA. The fact that genes involved in skeletal development (e.g. *FRZB* and *TNFRSF11B*, but also *OSTF1*, *FGFR3*, and *IGFBP3*; Table S3) change during ongoing OA processes confirms the hypothesis that OA chondrocytes lose their maturational arrested phenotype, specific for articular cartilage, towards their end-stage differentiation, resembling growth plate during skeletal development [3].

As reviewed by Barter and Young [31], gene expression differences in OA affected tissues may originate from changes in epigenetic control mechanisms. More recently, a comparison between the methylome of hip OA cartilage with cartilage of non-OA hips indeed showed more than 5000 differentially methylated loci whereas the annotated genes were mainly involved in pathways related to skeletal development [32] similar to the current and previous transcriptomic analyses [7]. Although direct association between such changes in DNA methylation and respective gene expression remains to be demonstrated, the skeletal developmental processes appear to consistently mark ongoing OA pathophysiology.

Recently, a GWAS for hand OA identified a locus in the aldehyde dehydrogenase 1 family, member A2 (*ALDH1A2*) gene [33]. Expression of *ALDH1A2* was shown to be allele dependent and with decreased expression in OA affected cartilage. Despite this and other recent successes of genome wide association studies [24], [28] a variety of the identified signals indicate chromosomal regions without obvious OA candidate genes or regions of high linkage disequilibrium with many relative unknown genes [24], [28]. Here, we provide a means of exploring the overall expression and behavior during disease in cartilage. Although OA should be considered a ‘whole joint disease’ [2] and expression profiles of other OA affected joint tissues such as those performed recently in subchondral bone [34] are highly valuable, expression profiles in OA cartilage could serve as one of the selection criteria to prioritize genes for functional follow-up studies and research directed at understanding pathophysiological mechanisms of OA and drug design. In our cartilage dataset, we found differential expression for several of the genes, among which *PAPPA* was most significant (*P* = 1.1×10^−6^), positionally localized in close neighborhood of one the arcOGEN genome wide hits: rs4836732 within the *ASTN2* gene. The exact linkage disequilibrium across this locus needs to be further explored. We also found *HBP1,* at the chr7q22 locus, to be differently expressed, although with small effect size in the OA versus preserved comparison (1.1-fold higher in OA affected cartilage). When comparing diseased cartilage (OA affected as well as macroscopically intact cartilage) with healthy cartilage we observed a much stronger and opposite direction of effects: healthy versus OA and healthy versus preserved both showed 1.4-fold lower expression (Table S4) in accordance with a previous study by Raine *et al.* showing increased expression of *HBP1* in OA affected cartilage [35]. Given that *HBP1* resides in the 7q22 gene cluster [24] results mark this gene as most likely candidate for further functional follow-up investigations.

Although *MCF2L* (MCF.2 cell line derived transforming sequence-like), a gene previously identified in GWA as an OA susceptibility gene [22], was not well-detected in the microarray analysis, the significant increased expression of neuronal growth factor (*NGF*) is worth mentioning in this respect. Neurotrophin-3 (NT3), another member of the NGF-family of proteins, enhances migration of premyelinating Schwann cells via Dbs/MCF2L [36], possibly implicating nociception in OA. Interestingly, antibodies generated against NGF or its receptor have been used successfully to treat OA patients and effectively reduced their pain [37]. The fact that *NGF* was not identified previously by comparing healthy with OA affected cartilage [7], [8] suggests that *NGF* may be more specific for the “late” OA process. Alternatively, selection of druggable targets from early-responsive genes that start changing before damage is irreversible could be more eligible to effectively slow-down or stop the OA process.

The sample collection is performed by well-trained lab personnel, however, we cannot exclude the possibility of minor contamination with bone tissue. In this respect, it is of note that several cartilage-specific genes (e.g. decorin or *DCN*, collagen type 2 A1 (*COL2A1*), cartilage intermediate layer protein (*CILP*), and cartilage oligomeric matrix protein (*COMP*) were amongst the 100 genes with highest levels of expression in the dataset while no bone-specific genes (e.g. *COL1A1, COL1A2*, *TNFRSF11B*, and bone sialoprotein II or *IBSP*) were identified here.

In conclusion, our results add to the insight into the ongoing pathological processes in OA cartilage by the identification of different gene expression patterns depending on OA severity as determined by Mankin score. This large scale analysis of joint-matched OA affected and preserved cartilage seems to hint at relatively consistent changes in gene expression during OA development. We think research and development of OA treatment could benefit by focusing on these similarities in gene expression changes and/or pathways.

## Supporting Information

Figure S1 **Typical example of hip (A) and knee (B) joint with areas of macroscopically preserved (arrow head) and OA affected cartilage (arrow; white scale bars indicate 500 mm).** Insets show detail of preserved (right) and OA affected area (left), in A separated by a dashed line (scale bar inset in B: 250 mm).
(TIF)

Figure S2 **Individual box plots per status for genes validated by RT-qPCR.**
(TIF)

Figure S3 **Relative changes in gene expression levels in preserved and OA affected cartilage relative to healthy cartilage for the 19 genes with at least 2-fold difference in the OA versus preserved analysis (note that the line does not imply continues changes given the fact that the healthy cartilage was derived from independent donors).**
(TIF)

Table S1 **Characteristics of OA donors included in the microarray analyses (discovery) and in the replication and characteristics of the healthy donors included in the microarray analysis.**
(XLSX)

Table S2 **Taqman probes used in the fluidigm RT-qPCR experiment.**
(XLSX)

Table S3 **Genes significantly differently expressed between OA and preserved cartilage in microarray analysis of 33 paired OA affected and preserved samples (FC: fold change; Pval: P-value; highlighted in yellow the genes that are also significantly different in the healthy versus preserved cartilage comparison).**
(XLSX)

Table S4 **Genes significantly differently expressed between preserved and healthy cartilage (FC: fold change; Pval: P-value).**
(XLSX)

Table S5 **Results of the validation of the genes with at least 2-fold significant differential expression between OA affected and preserved cartilage in the microarray analyses (Dir: direction of effects; FC: fold change; Pval: P-value).**
(XLSX)
