# Supplementary figures and images for: Genes Involved in the Osteoarthritis Process Identified through Genome Wide Expression Analysis in Articular Cartilage; the RAAK Study
Source: PLoS One. 2014 Jul 23;9(7):e103056. doi: 10.1371/journal.pone.0103056 (PMC4108379; doi:10.1371/journal.pone.0103056)

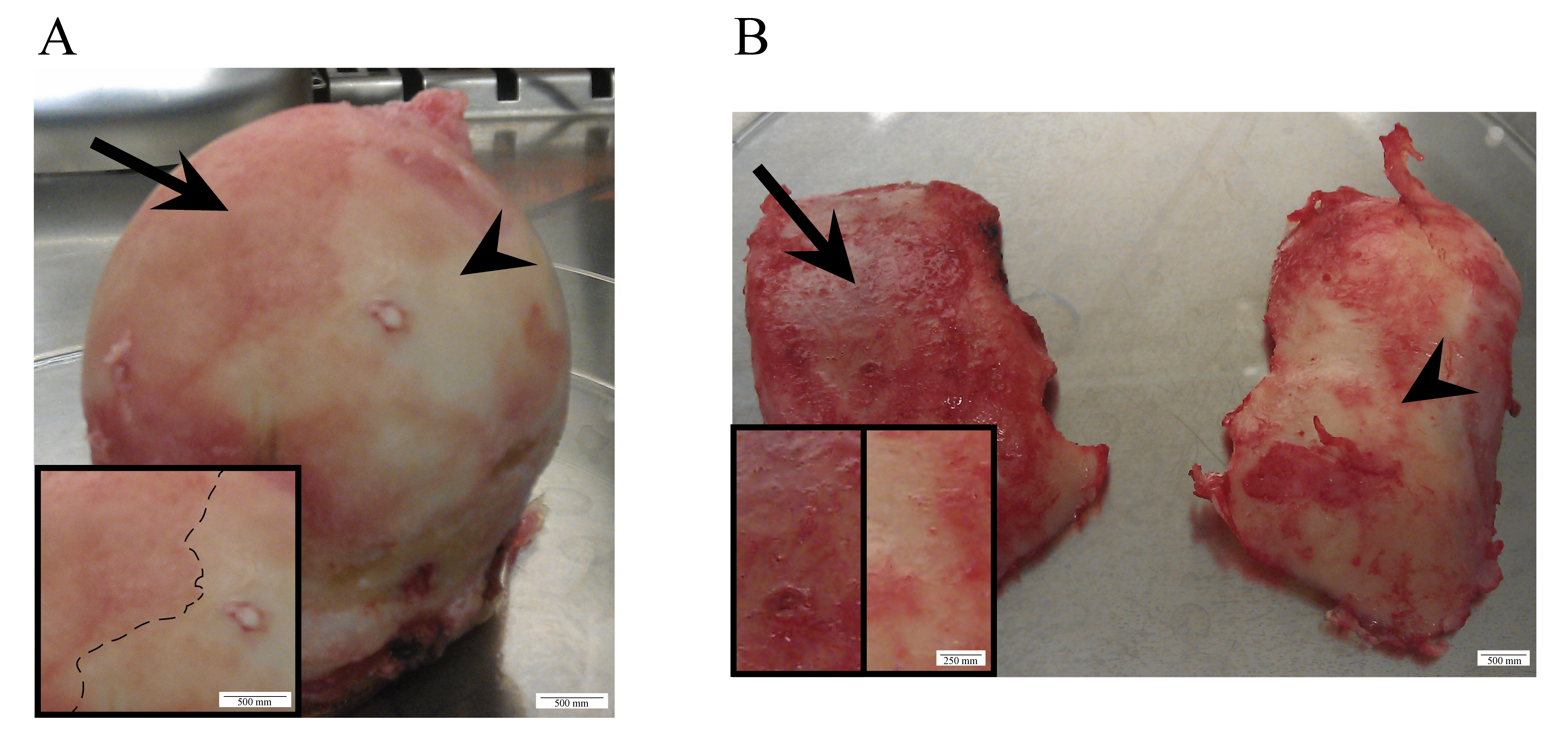

Supplement: Figure S1 — Typical example of hip (A) and knee (B) joint with areas of macroscopically preserved (arrow head) and OA affected cartilage (arrow; white scale bars indicate 500 mm). Insets show detail of preserved (right) and OA affected area (left), in A separated by a dashed line (scale bar inset in B: 250 mm). (TIF) [file pone.0103056.s001.tif]

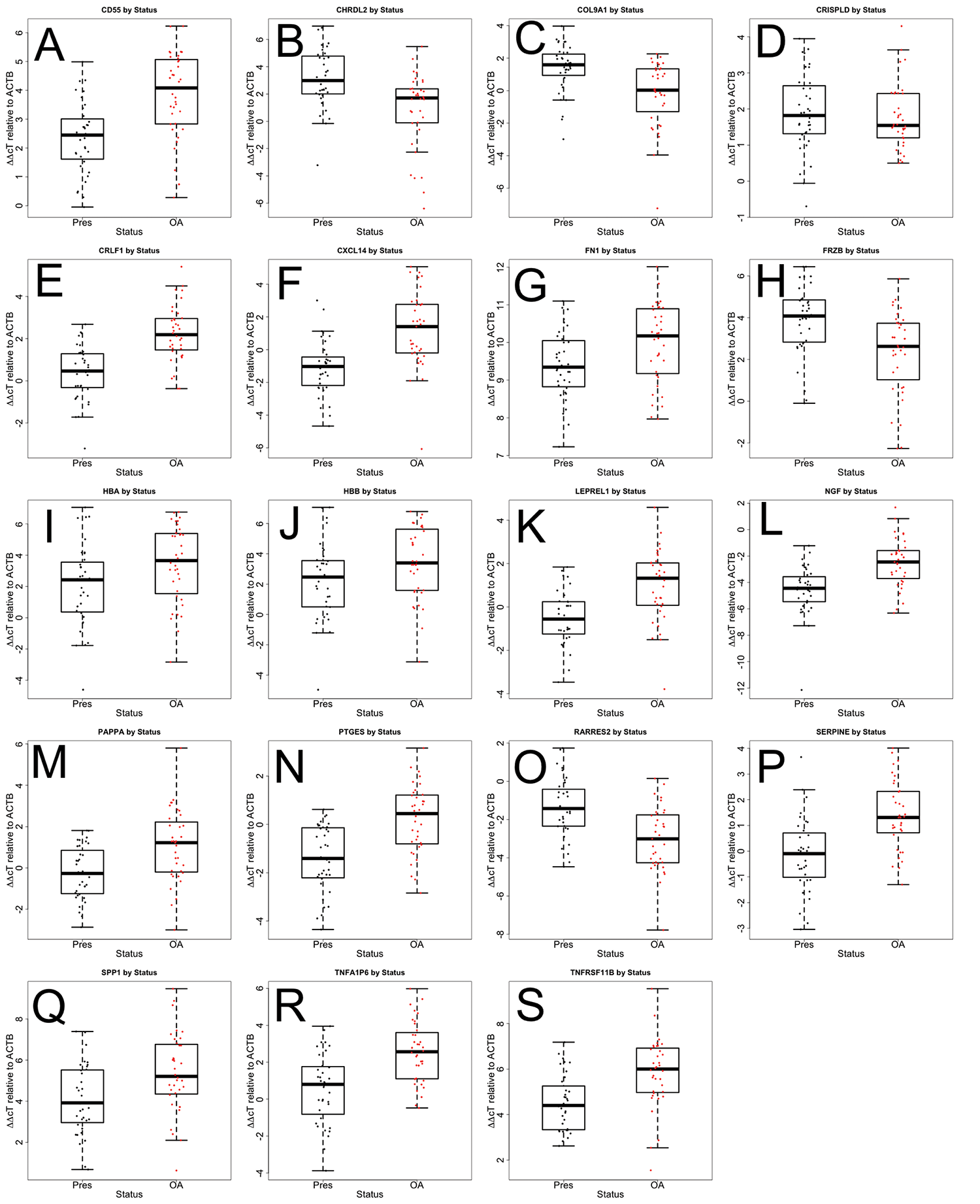

Supplement: Figure S2 — Individual box plots per status for genes validated by RT-qPCR. (TIF) [file pone.0103056.s002.tif]

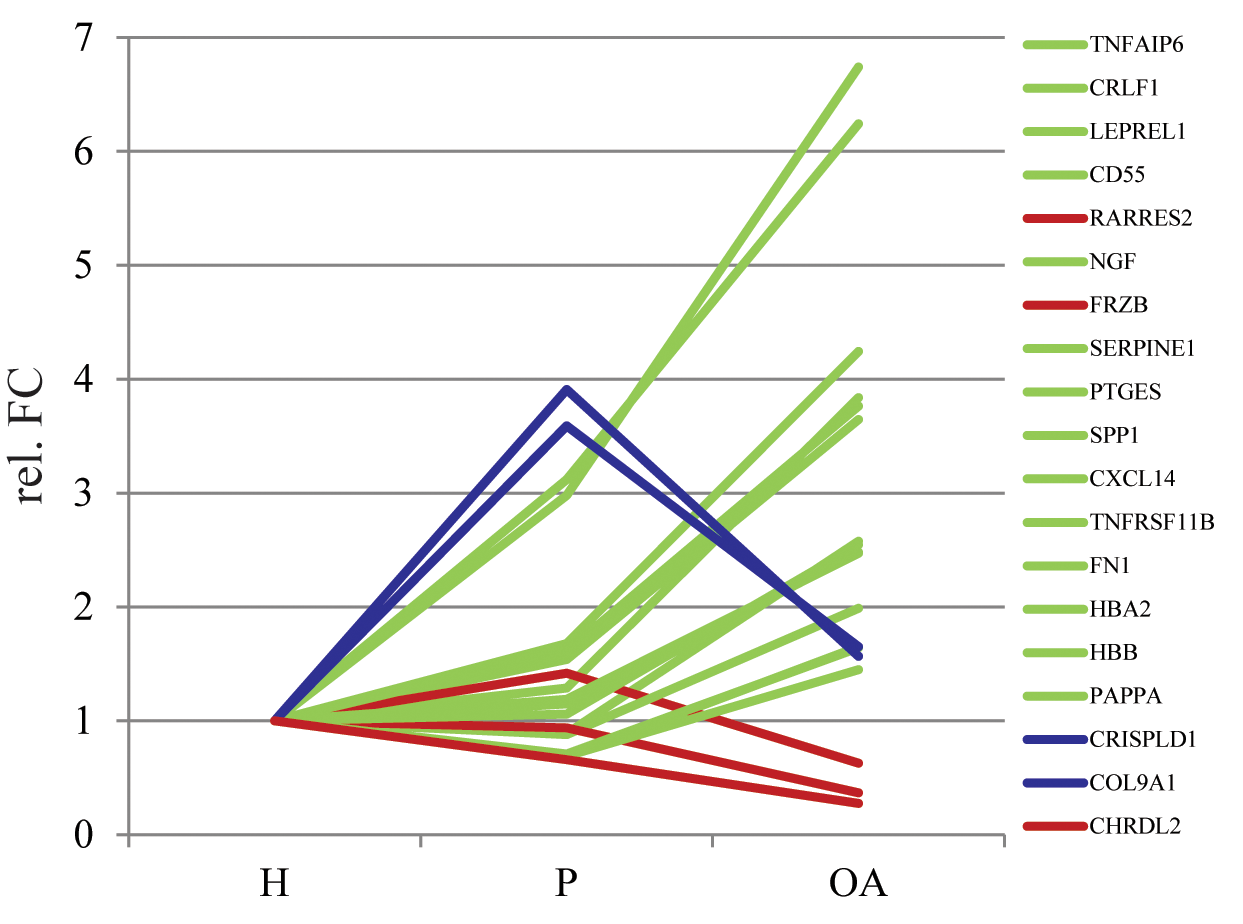

Supplement: Figure S3 — Relative changes in gene expression levels in preserved and OA affected cartilage relative to healthy cartilage for the 19 genes with at least 2-fold difference in the OA versus preserved analysis (note that the line does not imply continues changes given the fact that the healthy cartilage was derived from independent donors). (TIF) [file pone.0103056.s003.tif]
